# Methamphetamine and Ovarian Steroid Responsive Cells in the Posteriodorsal Medial Amygdala are Required for Methamphetamine-enhanced Proceptive Behaviors

**DOI:** 10.1038/srep39817

**Published:** 2017-01-03

**Authors:** Katrina M. Williams, Jessica A. Mong

**Affiliations:** 1 Program in Molecular Medicine, Department of Pharmacology, University of Maryland, Baltimore, MD, USA; 2 Department of Pharmacology, Program in Neuroscience, University of Maryland, Baltimore, MD, USA

## Abstract

Methamphetamine (Meth) is a psychomotor stimulant strongly associated with increases in sexual drive and impulse in both men and women. These changes in sexual motivation have a greater impact on women due to their likelihood of facing the greater burden of unplanned pregnancies, as well as increased risk for psychiatric co-morbidities such as depression. We have previously established a rodent model of Meth-induced increases in sexual motivation. Using this model, we have identified the posteriodorsal medial amygdala (MePD) via excitotoxic lesion studies as a necessary nucleus in Meth-facilitated female sexual motivation. While lesion studies give us insight into key nuclei that may be targets of Meth action, such an approach does not give insight into the identity of the specific MePD neurons or neural circuitry involved in Meth-induced increases in proceptive behaviors. Using the DAUN02 inactivation method, a recently established technique for removing behaviorally relevant cell populations, we present evidence that the ovarian steroid/Meth responsive cells in the MePD are necessary for Meth-induced facilitation of proceptive behaviors. These findings form the basis for future work that will allow for the classification of neuronal subtypes involved in the MePD’s modulation of proceptive behavior as well as a stronger understanding of the neurocircuitry of female sexual motivation.

Methamphetamine (Meth) is a potent psychostimulant drug of abuse that has a high propensity for increasing sexual drive^1^. Meth users report, among other effects, increased sexual desire, arousal, and risky sexual behaviors when using the drug^2^,^3^. Like men, women report that Meth use heightens their sexual experiences and is associated with increased sexual drive and activities^1^,^2^,^3^,^4^. Unlike men, women are at a greater disadvantage due to the psychological and socioeconomic burdens^5^,^6^,^7^. High-risk sexual behaviors not only perpetuate sexually transmitted diseases such as HIV/AIDS, but also lead to increases in unplanned pregnancies^1^. Despite the significant health and socioeconomic concerns, the neural mechanisms underlying this drug–sex association are not well understood.

Several laboratories, including our own, have demonstrated that Meth facilitates sexual motivation in female rodents^8^,^9^,^10^,^11^. In the female rat, a component of sexual motivation is characterized by proceptive behaviors (hops, darts, solicitations, ear wiggling) that are exhibited by a receptive female to stimulate the male to copulate^12^. In our established model, administration of Meth to sexually receptive females (ovariectomized females treated with estradiol and progesterone; EB + P) markedly increases proceptive behaviors compared to EB + P treatment alone^8^,^9^,^10^. This Meth-induced increase in female sexual motivation is specific to sexually relevant stimuli as Meth-treated females display increases in proceptive behavior toward intact males and androgen-treated castrate males but not toward castrate males^13^. Moreover, our work suggests a neurobiological correlate that underlies Meth-induced increases in proceptive behaviors. In sexually naïve females, the combined administration of Meth and EB + P increases posteriodorsal medial amygdala (MePD) neuronal activation as measured by cFos immunopositive cells^8^. Excitotoxic lesion of the MePD blocks the Meth-induced increases in proceptive behavoir suggesting that the MePD is an important drug-sex nexus^10^. While lesion studies are helpful in determining the potential role the targeted brain region to a given behavior or physiological process, the lesion is often imprecise and does not allow for the specific targeting of functionally relevant neuronal cell types or ensembles necessary for the output behavior.

Recently, Bruce Hope and colleagues have pioneered a technique that allows for determining whether specific neuronal ensembles are required for a specific behavior^14^,^15^. Using Sprague Dawley rats expressing a cFos-lacZ transgene, the ‘DAUN02 inactivation technique’ exploits the expression of β- galactosidase (β-gal) under the cFos promoter to remove neurons that are activated by a specific stimulus or state while the neighboring non-activated neurons remain intact. The prodrug DAUN02 is converted by β-gal to daunorubicin within the neurons that are expressing β-gal resulting from the activation of the cFos promoter. Daunorubicin is toxic to the cells expressing β-gal resulting in apoptosis^16^,^17^,^18^ and thus a cell-specific lesion. As β-gal expression is induced only in neurons with a strong activation of the cFos promoter and not in Fos negative or weakly activated neurons^14^,^19^, this method is a powerful approach for testing whether activation of specific neuronal groups or ensembles are necessary for an associated behavior^14^,^15^,^19^.

In the current study, we have adapted this model to test whether the Meth/EB + P responsive cells (as measured by cFos expression) in the MePD are necessary for the associated increase in proceptive behaviors. In the present study, our goals are 1) to establish that cFos-lacZ transgenic rats exhibit Meth-induced MePD neuronal activation as well as increases in proceptive behaviors and 2) to demonstrate that Meth/EB + P responsive MePD neurons constitute a behaviorally relevant neuronal ensemble required for the Meth-facilitated increases in proceptive behaviors by specifically removing these neurons with DAUN02 inactivation. Once established, future studies will work to elucidate the cell types and circuitry associated with the behavioral modulation.

## Material and Methods

### Animals

cFos-lacZ transgenic Sprague Dawley rat^20^ (275–300 g; n = 45) breeding pairs were obtained by Friedbert Weiss, Ph.D. (The Scripps Research Institute (TSRI)) and were bred in-house and housed in the Laboratory Animal Facility of the Health Sciences Facilities at the University of Maryland, School of Medicine under a reversed 12 h:12 h dark:light cycle (lights off at 0900 h) with food and water available ad libitum. Transgenic pups and their wild-type littermates were genotyped to verify expression of the c-Fos-β-galactosidase fusion protein by taking ear punches or tail segments from pups at the time of weaning. DNA was isolated from samples using isopropanol precipitation. Briefly, tissue was incubated overnight in proteinase K lysis buffer at 55 °C, DNA was precipitated with isopropanol from solution and added to a 70% ethanol solution and pelleted. DNA is then amplified using OneTaq kit (New England Biolabs) using 400 μg DNA, 10 micromolar forward and reverse primers (IDT, Iowa). Primer sequences were LacZf2538: 5′-GTT GCA GTG CAC GGC AGA TAC ACT TGC TGA-3′ and LacZR2926: 5′-GCC ACT GGT GTG GGC CAT AAT TCA ATT CGC -3′. Samples were run on a 2% agarose gel using tris-acetate (TAE) buffer and ethidium bromide. LacZ bands are visualized under UV light at 388 bp.

All animals were group housed until cannula placement when they were placed in single-housed conditions. All animals were bilaterally ovariectomized under isoflurane anesthesia and allowed a 7–10 day recovery period following surgery. All procedures were approved by the University of Maryland, Baltimore Institutional Animal Care and Use Committee and were in accordance with the National Institutes of Health Guide for Care and Use of Laboratory Animals. All efforts were made to minimize animal suffering and to reduce the number of animals used.

### Ovarian steroids and Meth Treatment

**Figure 1:**
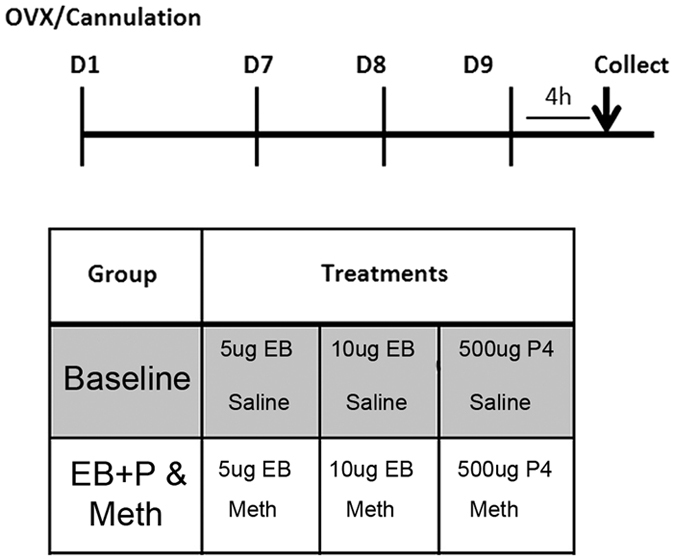
Standard treatment paradigm for Meth and steroid hormones (EB + P). Animals are OVX and allowed one week to recover. Days 7–9 post-operatively, animals receive saline or Meth and EB + P injections.

All animals were treated with estradiol, progesterone, and either saline or Meth following the treatment procedures used in our previous studies^8^,^9^,^10^ (Fig. 1). Our treatment paradigm is as follows: Forty-eight hours before the start of the experimental assay the animals were administered 5 μg 17-β-estradiol benzoate (EB; Sigma Aldrich, St. Louis, MO) in sesame oil subcutaneously (SC) followed by 10 μg EB or 0.1 mL 24 h later. Four hours prior to experimental assay, the rats were injected with progesterone (P, 500 μg, SC; Sigma-Aldrich). During the three days of hormonal priming rats received a daily injection of Meth (5 mg/kg/day, IP; Sigma-Aldrich) or saline. This dose and administration protocol of Meth was previously demonstrated to facilitate female sexual motivation and behaviors without causing an increase in stereotypy or general locomotion at the time point of the experimental assay^8^,^9^.

### Statistical analysis

All results are expressed as means ± SEM. One-way or two-way ANOVAs followed by Tukey’s post hoc tests were used and are indicated where appropriate. Student’s t tests were used for two-group comparisons. Significance threshold was set at p ≤ 0.05. All statistical tests were conducted using GraphPad Prism software (version 6, San Diego, CA USA).

### Beta-Galactosidase and Fos immunoreactivity in the MePD following Meth and ovarian steroid treatment in cFos-lacZ transgenic female rats

Previous work has shown that in wild-type Sprague Dawley rats, Fos expression is highest following the combined administration of Meth and EB + P at the time of behavior (4 hours post-treatment). To verify that cFos-lacZ transgenic rats exhibited a similar response, protein expression of cFos and its proxy, β-gal were examined. Four hours after the last Meth and hormone injection (see Fig. 1), animals were transcardially perfused under ketamine/xylazine anesthesia (98 mg/kg and 18.5 mg/kg, respectively; IP) with 0.9% saline containing sodium nitrate, followed by 4% paraformaldehyde in 0.5 M kPBS. Following perfusion, the brains were extracted, post-fixed, cryoprotected, frozen, and processed for immunohistochemistry. Briefly, cohorts containing sections from all treatment groups were rinsed in kPBS, blocked in 0.5% Triton-X, 5% normal goat serum, and 1% hydrogen peroxide in kPBS for 1 hour. Sections were incubated overnight at 4 °C with a mouse monoclonal antibody targeted to beta-galactosidase (G8021 Clone GAL-13 Sigma, St. Louis, MO) at a dilution of 1:30000 or 48 h at 4 °C with a rabbit polyclonal antibody targeted to cFos (Cat No. 18-0172 Calbiochem Invitrogen, Carlsbad, CA) at a dilution of 1:40,000 in 0.05% Triton and 1% normal goat serum in kPBS. Sections were then washed and incubated in a secondary antibody (goat anti-mouse or goat anti-rabbit, respectively; Vector Laboratories, Burlingame, CA) in 0.5% Triton in kPBS for an hour, followed by avidin-biotin complex. Sections were developed in DAB (3-3′-Diaminobenzidine tetrahydrochloride, 04001-5 Polysciences, Inc., Warrington, PA). Sections were mounted serially on 2% gelatin-coated glass slides and cover slipped. The number of β-gal- and cFos-immunoreactive cells were counted with the aid of the Neurolucida software (MicroBrightField, Colchester, VT) using laboratory standard methods in accordance with previously defined parameters for the MePD^8^,^9^. Briefly, a standardized contour was used to demarcate the MePD. Three brain sections (in series) separated by 140 μm were used. In the event that three sections from the appropriate brain region could not be obtained, the animal was excluded from that region’s analysis. Both sides of this bilateral nucleus were included in the analysis, resulting in six counting contours. From these six contours, average β-gal or cFos- positive cell number per section for each region was derived. Both β-gal and cFos were evaluated in each animal to determine if expression levels followed similar patterns and to make a more direct comparison to cFos expression from previous studies. Slides were anatomically matched and numerically coded so that the investigator conducting analysis was blinded to the experimental group.

### Meth-induced increases in proceptive behavior in cFos-lacZ transgenic female rats

Our previous studies consistently demonstrate that Meth increases proceptive behavior on average 2.5 fold in EB + P treated Sprague Dawley rats^8^,^9^,^10^. This established behavioral paradigm was applied to the Lac Z+ transgenic rats to verify similar behavioral responses compared to wild types. The behavioral tests were conducted under dim red light in the dark phase of the light cycle between 1300 and 1600 h, approximately 4 h after the last Meth injection (Fig. 1). On the day of behavioral testing, two sexually naïve Sprague Dawley males were each placed alone in a 50 cm × 38 cm × 25 cm Plexiglas observation chamber and allowed to acclimate over 5 minutes. An experimental female was then placed with each male. Each behavioral test was recorded by a video camera and was completed when 15 min had elapsed. Between behavioral tests, males were allowed 5 minutes to rest before the next encounter, and each female was exposed to both males. Naïve males were chosen for these tests as less vigor encouraged a reduction in rejection behaviors seen. During the tests, the investigator remained at a consistent location approximately 0.5 m away from the observation chambers during all trials.

An experimenter blind to the treatment groups scored the proceptive sexual behaviors as previously described^8^,^9^,^10^. In the rat, female-initiated “courting” has been classically termed proceptive behavior^12^. These behaviors consist of hops, darts, solicitation postures and ear wiggling to acquire the male’s attention. Briefly, the number of proceptive behaviors, receptive behaviors (lordosis score, quotient), rejection behaviors from the females, and male mounts that occurred in 15 min were quantified. To control for any differences in male behavior, female behaviors were averaged between the two 15 minute sessions. Male mounting was compared between individual animals (male vs. male) as well as between female treatment groups.

### DAUN02 Inactivation of Meth- and EB+ P-active cells in the MePD

The DAUN02 paradigm occurred over an approximately three-week period, starting with the initial surgeries. The paradigm is then broken into three main phases as described below.

#### Stereotaxic surgeries targeting posteriodorsal medial amygdala

Animals were placed in a stereotaxic apparatus (Kopf Instruments, Tujunga, CA) under isoflurane anesthesia; an incision was made to expose the skull. Chronic indwelling 25-gauge guide cannulae (Plastics One, Roanoke, VA) were bilaterally implanted into the MePD (3.0 mm posterior, ±3.55 mm lateral, and 7.0 mm ventral from Bregma) and affixed to the skull using dental acrylic and bone screws. Dummy stylets were placed in the guide cannulae in order to keep them unobstructed. Animals were OVX at this time and given a week to recover prior to the DAUN02 Lesion Phase.

### Lesion Phase

**Figure 2:**
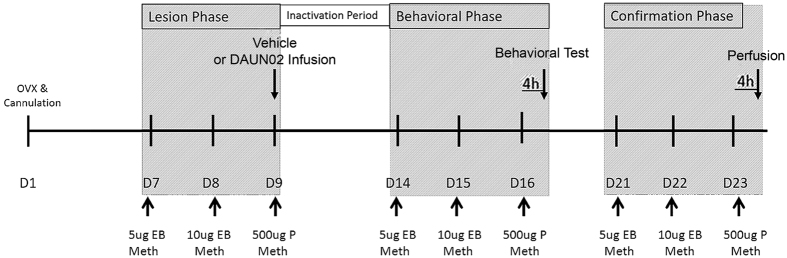
Extended treatment paradigm for animals receiving DAUN02 or vehicle infusions. Days 7–9 post-operatively, animals receive Meth and EB + P injections. At the time of progesterone administration on day 9, DAUN02 or DMSO vehicle is infused. There is a period to allow for the lesion to occur and DAUN02 to be cleared from the region. The following week, animals receive a second round of hormones and Meth and have behavior evaluated four hours following progesterone. A third round of EB + P and Meth is then admistered in order to evaluate cFos-ir four hours following EB + P and Meth after the single DAUN02 infusion three weeks prior.

Animals were treated as described in Fig. 2. All animals received infusions of DAUN02 (HY-13061, MedChemExpress, Princeton, NJ, USA) or vehicle 10 minutes prior to progesterone and Meth injections. During the microinfusions, the dummy stylets were removed and replaced by 33 gauge microneedles that project 1.3 mm below the guide cannulae and were attached via polyethylene tubing to a 25 μl Hamilton syringe (700 series, Hamilton, Reno, NV). Fluid flow rate was controlled by a BASi Bee pump attached to a Bee Hive controller (Bioanalytical Systems, Inc., West Lafayette, IN). Bilateral lesions targeting the beta-galactosidase producing cells of the MePD were produced with injections of 0.5 μl of DAUN02 (4.0 μg/μl in 5% DMSO PBS). Infusions occurred over 5 min and the microneedles remained in place for an additional 5 min to ensure diffusion away from the needle tips. Control lesions were performed using the same methods, but using vehicle injections.

Initial pilot studies infused DAUN02 over the first three days of Meth exposure. Optimization of the study led to a reduction of this protocol to a single injection on day 9 (Fig. 2). Triple infused animals were compared to singly treated animals and no statistical difference was found; therefore, the groups were collapsed. All animals were given 5–7 days for the DAUN02 to lesion the cells and to be adequately metabolized before entering the next phase.

### Behavioral Phase

All animals were treated with Meth and EB + P as described in Fig. 2 and tested for sexual behavior as described above four hours after receiving the combined Meth and P treatment.

### Confirmation Phase

#### Cannula Placement

The animals were transcardially perfused under ketamine/xylazine anesthesia (60 mg/kg, IP) with 0.9% saline containing sodium nitrate, followed by 4% paraformaldehyde in 0.5MkPBS. Brains were stored overnight in a 4% paraformaldehyde solution and then cryoprotected in 30% sucrose in kPBS. After cryoprotection, the brains were frozen on dry ice and stored at −80 °C until processed for Nissl staining. Brains were sectioned (35 μm) in the coronal plane in a cryostat and stored in a cryoprotectant solution (ethylene glycol/glucose in sodium phosphate buffer) until processed. The sections were rinsed in kPBS and mounted serially on 2% gelatin-coated glass slides. They were rehydrated in dH2O, stained in a 0.5% Cresyl Violet solution containing 1 M sodium acetate, and 1 M acetic acid and coverslipped. A placement was deemed appropriate when the cannula track was located within sections that corresponded to plates 54–60 in a standard brain atlas^21^ and fell dorsolateral to the optic tract.

#### Confirmation of cFos reduction

Brains collected for cannula placement verification also underwent cFos immunohistochemistry. Briefly, during the confirmation phase as seen in Fig. 2, animals were retreated with both Meth and hormones and collected four hours after progesterone treatment on day 23. cFos IHC was completed as described above to determine if fewer cells expressed cFos, during the time of behavior, after DAUN02 infusions.

## Results

### Administration of Meth increased Fos- and Beta Galactosidase-immunoreactivity in the MePD of hormonally primed cFos-lacZ transgenic rats

**Figure 3:**
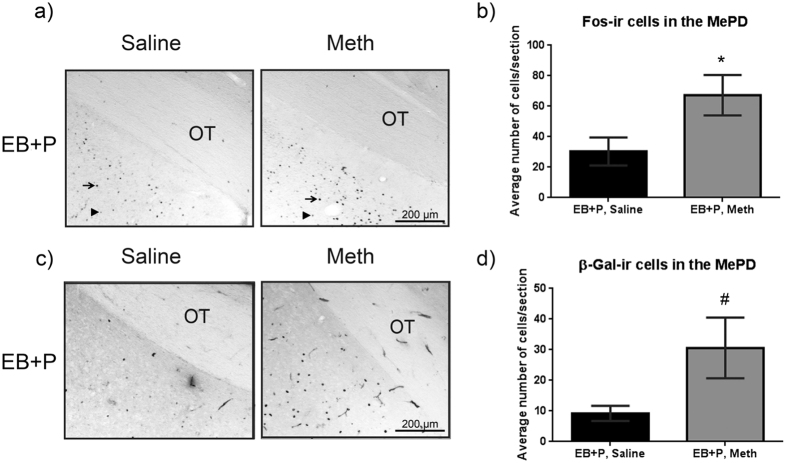
Effects of Meth on cFos immunoreactivity (Fos-ir) & Beta-galactosidase immunoreactivity (β-Gal-ir) in the posteriodorsal medial amygdala. Adult OVX cFos-lacZ transgenic rats were primed with EB + P and received either saline or Meth (5 mg/kg). Four hours after the final injection, tissue was collected for cell counts. (**a**) The photomicrographs represent the Fos-ir in the MePD. Arrows identify highly expressed cFos-ir and arrowheads identify low expressing cFos-ir. Both expression types were included in quantification. (**b**) Quantification of cFos-ir in the MePD. A t test revealed a significant difference between groups (t_(4)_ = 2.29, *p < 0.05, n = 3). (**c**) The photomicrographs represent the β-Gal-ir in the MePD. (**d**) Quantification of β-Gal-ir in the MePD. A t-test shows a strong trend for increase in the presence of Meth (t_(4)_ = 2.09, ^#^p = 0.05, n = 3). OT = optic tract.

Similar to our previously published data on Sprague-Dawley wild-type females, the number of cFos-immunoreactive (-ir) cells increased in the MePD following Meth administration in the cFos-lacZ transgenic females (Fig. 3a). This is quantified in Fig. 3b, which shows approximately 2-fold increase in Fos-ir after the administration of Meth/EB + P, compared to saline/EB + P-treated controls (t_(4)_ = 2.28, p < 0.05). Similarly, the number of β-gal-ir cells in the MePD of the cFos-lacZ rats increased following Meth administration to hormonally primed females (Fig. 3c). This was quantified to be a 3-fold increase after the administration of Meth/EB + P (t _(4)_ = 2.09, p = 0.05; Fig. 3d); however, the number of β-gal -ir cells in the MePD was notably fewer than the Fos-ir cells in both treatment groups.

### Meth administration increases proceptive behaviors in hormonally primed cFos-lacZ transgenic rats

**Figure 4:**
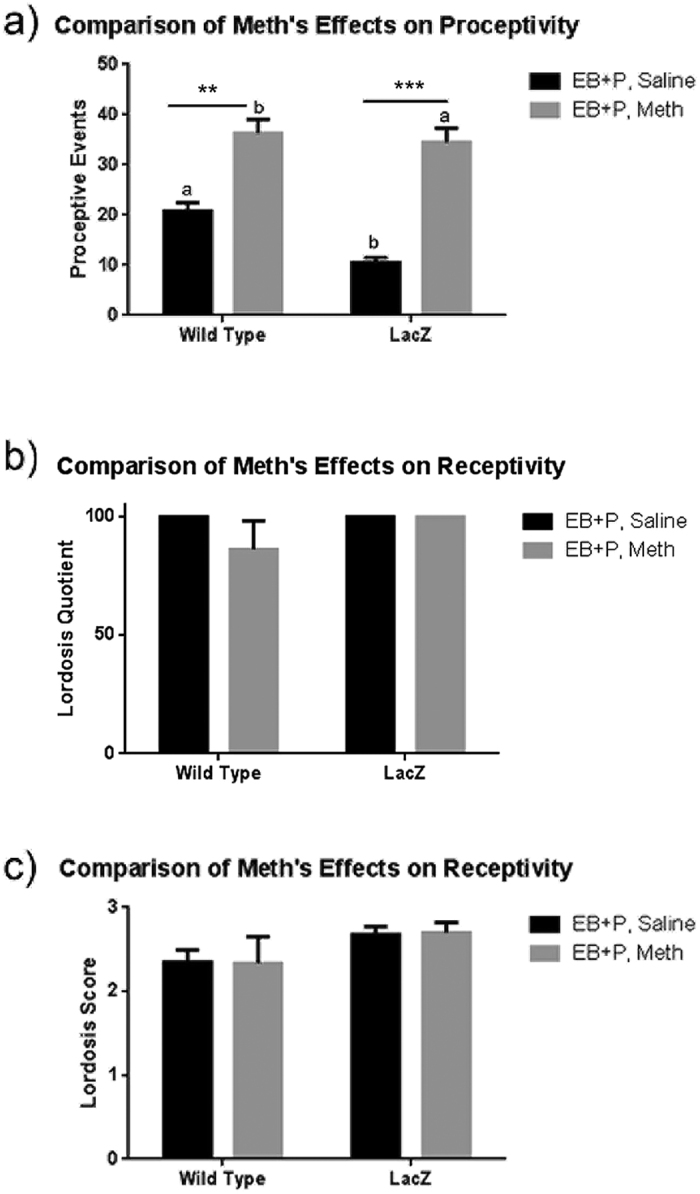
Effect of Meth on proceptive behaviors (**a**) and lordosis (**b**,**c**) in hormonally primed female rats: a comparison between wild type and cFos-lacZ rats. Adult OVX WT Sprague-Dawley and cFos-lacZ transgenic rats were primed with EB + P and received either saline or Meth (5 mg/kg). Behavioral tests were performed four hours after the final injection. A two-way ANOVA demonstrates there is a main effect of Meth (F_(1,12)_ = 61.99, p < 0.0001, n = 3–5; **a**) and Strain (F_(1,12)_ = 5.75, p < 0.05, n = 3–5; **a**) on proceptive behavior. A Tukey’s post-hoc multiple comparison test reveals that the saline treated animals are significantly different from their respective Meth treated group (WT: **p < 0.005, LacZ: ***P < 0.0005) and the saline and hormone treated animals are significantly different from the other strain’s Meth treated group (^a^p < 0.01, ^b^p < 0.0001), but each saline treated or meth-treated pairs are not different from each other. There was no effect of drug or strain for either lordosis quotient (F_(1,13)_ = 1.46, F_(1,13)_ = 1.46, **b**) or lordosis score (F_(1,14)_ = 0.001, F_(1,14)_ = 2.91, **c**).

Meth significantly increased proceptive behaviors in hormonally primed wild type and cFos-lacZ transgenic rats compared to those treated with EB + P alone. A two-way ANOVA with drug and genetic strain as factors revealed a significant main effect of Meth (F_(1,12)_ = 61.99, p < 0.0001; Fig. 4a). A Tukey’s post hoc test indicated that Meth increased proceptive behaviors in both WT (p < 0.005) and cFos-lacZ transgenic rats (p < 0.0005) compared to their respective saline controls. The two-way ANOVA additionally revealed a significant main effect of strain (F_(1,12)_ = 5.75, p < 0.05; Fig. 4a). A Tukey’s post hoc test indicated that the difference between strain resulted from each saline control being significantly different from the other strain’s Meth-treated group (p < 0.01, p < 0.0001). There was no effect of drug or strain for either lordosis quotient (F_(1,13)_ = 1.46, F_(1,13)_ = 1.46, Fig. 4b) or lordosis score (F_(1,14)_ = 0.001, F_(1,14)_ = 2.91, Fig. 4c).

### Meth induction of cFos-ir cells in the MePD decreased following a single DAUN02 infusion

**Figure 5:**
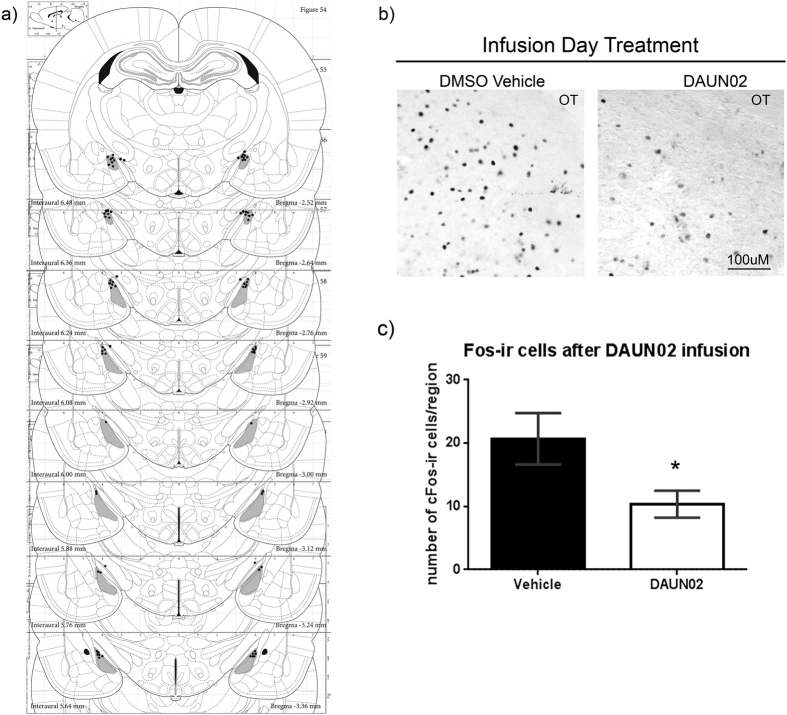
Effect of DAUN02 infusions on cFos-ir in the MePD. (**a**) Schematic reconstruction of the cannula placements in the MePD (cannula ends are marked in black, any black circle outside of the nucleus as defined in grey is considered a “miss”). Coronal sections through the medial amygdala, adapted from Paxinos and Watson^21^. Animals with cannula placement outside of the MePD were not included in behavioral analysis. (**b**) Photomicrographs represent cFos in the MePD after Meth and hormone administration following DAUN02 or vehicle infusions. (**c**) A student’s t-test showed DAUN02 significantly reduced Fos-ir cells in the MePD compared to the vehicle-treated cohort (t_(15)_ = 2.163, *p < 0.05, n = 9). OT = optic tract.

Following DAUN02 infusion and behavioral testing, tissue was evaluated for the placement of the cannulae (Fig. 5a) and number of cFos positive cells (Fig. 5b and c) four hours following the final administration of Meth and EB + P. Any animals that had a cannula placement outside the MePD (as shown in grey in Fig. 5a) was not included in the analysis. Similarly, tissue was evaluated using Cresyl Violet Nissl stain to determine that the MePD was intact (Supplementary Figure). A student’s t-test revealed that DAUN02 infusion resulted in a significant decrease in cFos-ir cells in the MePD compared to Vehicle-infusion (t_(15)_ = 2.163, p < 0.05; Fig. 5c).

### DAUN02-inactivation of Meth/EB + P-responsive cFos+ cells reduced Meth’s effects on proceptivity

**Figure 6:**
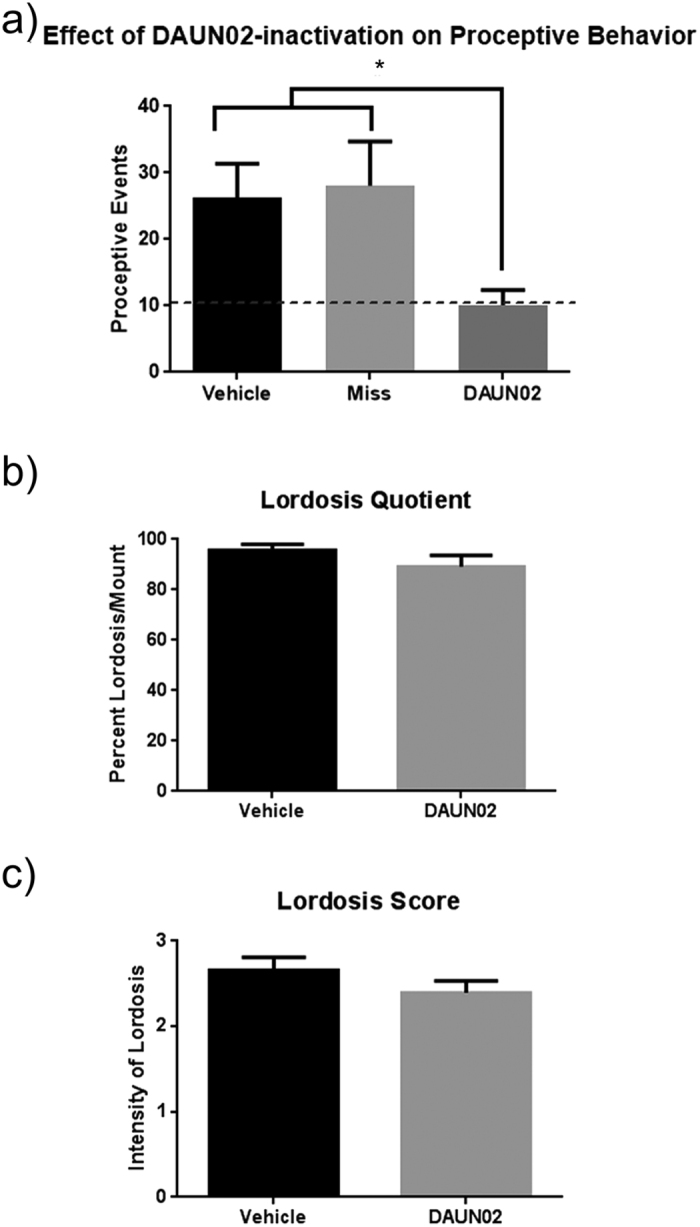
Effects of DAUN02 infused into the MePD on Meth-enhanced female sex behaviors. Following DAUN02 inactivation, animals were hormonally primed and treated with Meth (5 mg/kg) over three days. Behavioral assays were completed four hours following the final treatment. (**a**) A one-way ANOVA revealed DAUN02 significantly reduced proceptive sex behaviors in Meth and hormone treated animals (F_(2, 25)_ = 4.381, p < 0.05, n = 7–11). Tukey’s post-hoc comparison reveals that both vehicle-treated (n = 11), and animals with inappropriate cannula placement (miss, n = 7) show significantly greater numbers of proceptive behaviors than animals who had DAUN02 infused into the MePD (n = 10) (*p < 0.05). Dashed line shows EB + P-only baseline behavioral mean from Fig. 4a for comparison. (**b**), (**c**) Receptive Sex behaviors after DAUN02 infusion showed no significant differences between groups for Lordosis Quotient or Lordosis Score.

A one-way ANOVA revealed that DAUN02 infusions directly into the MePD of cFos-lacZ transgenic rats treated with Meth and EB + P reduced Meth-facilitated increases in proceptivity (F_(2,25)_ = 1.176, p < 0.05; Fig. 6a). A Tukey’s multiple comparison test indicated that DAUN02 treated rats exhibited significantly fewer proceptive behaviors, about 1.5-fold fewer, compared to the Vehicle infused controls (p < 0.05; Fig. 6a). Moreover, proceptive behaviors in DAUN02-infused animals that had improperly placed cannulae (i.e. not targeting the MePD) were not significantly different from the vehicle infused controls but did display significantly more proceptive behaviors than the properly targeted cohort (p < 0.05, Fig. 6a). Significant differences were not noted in the lordosis quotient or lordosis score (t_(14)_ = 1.34, p = 0.20, Fig. 6b; t_(14)_ = 1.25, p = 0.23, Fig. 6c).

## Discussion

While recent surveys suggest a national decline in Meth use, women from various socioeconomic and racial backgrounds show an upward trend in use^22^,^23^,^24^. Meth abuse in women is associated with high-risk sexual behaviors that raise significant health concerns over increased rates of sexually transmitted diseases such as HIV/AIDS and unplanned pregnancies among Meth-addicted women^1^,^25^. Self-reporting studies strongly associate Meth use with heightened sexual drives, desires and sexual activities in women^1^,^2^,^3^,^4^. While social cues specific to human interactions may in part contribute to heightened sexual drive and behaviors, mounting evidence from animal studies suggests a neurobiological correlate leading to changes in sex behavior after Meth exposure. The goals of the current study were to establish the Fos-lacZ transgenic rat and the DAUN02 inactivation method^14^,^20^ as a model system to better study the mechanisms through which Meth activates the neurocircuitry underlying sexual motivation. The results from the current study demonstrate (1) the Fos-lacZ transgenic rats expressing β-gal under the cFos promoter exhibited similar functional neuroanatomical and sexual behavioral responses to Meth as previously reported in wild-type Sprague Dawleys^8^ and (2) targeted DAUN02 removal of EB + P/ Meth responsive cells (i.e. those that are Fos-ir) in the MePD attenuated Meth-facilitated increases in proceptive behaviors.

Others and we have reported that Meth increases proceptive (or solicitational) sex behaviors in female rats^8^,^9^. In sexually naïve females, the combined administration of Meth and ovarian hormones increases neuronal activation of MePD neurons (as measured by cFos) over either one alone^8^. Moreover, this increase is present at time when females would be sexually receptive suggesting that the activated cells maybe mediating the increase in proceptive behavior. Indeed, excitotoxic lesions of the MePD eliminate Meth-induced increase in proceptivity suggesting a role for the MePD in mediating the Meth’s effect on proceptivity^10^. Results from the current study provide now provide more discrete evidence that population of MePD neurons that are activated by the administration of Meth and ovarian hormones play a significant role in mediating the Meth-induced increases in proceptivity. Thus, these Meth/EB + P responsive MePD neurons may represent a discrete neuronal ensemble responsible for the modulation of proceptivity in the presence of Meth.

In a series of reports, Hope and colleagues have recently established that the c-Fos-LacZ rats in combination with the DAUN02 inactivation technique is a suitable model for selectively eliminating specific neuronal ensembles and testing for their causal role in specific behavioral outputs^14^,^16^,^17^,^18^,^19^. The DAUN02 lesions are ideal for determining the role of specific cells, as they do not cause a global disruption of the parenchyma^14^. Instead, only those cells expressing high levels of Fos (and thus β-Gal) are removed via apoptosis^18^. Similar to what others have found in other regions, in sexually naïve animals, we observed fewer β-Gal-ir cells than Fos-ir cells within the MePD. While the potential exists that this is due to incomplete transmission of the transgene (perhaps in heterozygous animals), it has been shown that β-gal expression is induced only in neurons with strong expression of Fos^14^,^19^. Curiously, fewer cFos-ir cells were observed in the vehicle-treated controls for the DAUN02 inactivation phase (Fig. 5c; vehicle group) compared to the sexually naïve cohort treated with Meth and EB + P (Fig. 3b). This reduction in c-Fos-ir cells in the DAUN02 vehicle controls may be due to the repeated Meth exposure. In other brain regions, repeated or chronic Meth administration reduces the number of Fos-ir cells^26^,^27^. In the DAUN02 experiments, animals received nine Meth injections compared to three received by the sexually naïve animals.

Nevertheless, the number of Meth /EB + P responsive MePD cells (as measured by cFos-ir) were significantly reduced in the DAUN02 treated animals compared to the vehicle controls treated with Meth/EB + P. Furthermore, this reduction in cFos-ir cells was associated with attenuation of Meth-induced increase in proceptivity suggesting that the ensemble of MePD cells responsive to the combination of Meth and ovarian steroids is necessary for the increase in behavior. In further support for the specificity of the cell-targeted lesion, animals who received DAUN02, but who had improper cannulae placement outside of the MePD (i.e. “misses”) exhibited levels of proceptive behavior similar to that of the vehicle treated controls. While we did not observe a Meth-induced increase in the the lordosis response, this is not surprising as the combined treatment of EB + P produced a near maximal lordosis quotient and score. Taken together these findings support the hypothesis that Meth’s effects on proceptivity are dependent on a discrete population of neurons within the MePD, and that this ensemble is relevant specifically to the behavioral effects of Meth on proceptivity alone.

Of note, while some cFos-ir cells in the DAUN02 treated cohort remained, they were not sufficient to allow Meth-induced increases in proceptivity. These remaining cFos-ir cells may have escaped the cell-specific removal due to a DAUN02 induced inactivation that did not lead to cell death^18^. Nervertheless, the marked reduction in cFos-ir cells suggests that DAUN02 was effective in removing a signicant portion of the targeted cells. Alternatively, as repeated Meth exposure results in sensitization of the neural substrate^28^,^29^,^30^, MePD neurons that might have been low-responders to the Meth/hormone treatment during the lesion phase and thus not targeted may have become sensitized over the two subsequent rounds of exposure resulting in detectable levels of cFos following the third exposure.

Previous studies implicate the MePD as a key site for the modulation and enhancement of female sexual motivation^11^,^31^,^32^,^33^,^34^,^35^,^36^. The MePD processes and integrates sexually relevant signals such as chemosensory^37^ and somatosensory signals^38^,^39^,^40^,^41^,^42^, suggesting that the MePD may act as a nexus in the expression of motivated sexual behaviors. Lesions of the MeA lead to a decrease in lordotic responses^33^, proceptive behaviors^32^,^34^, and conditioned place preference^35^, whereas stimulation of the MeA increases receptive behaviors such as lordosis^33^,^34^. Our previous^8^,^9^,^10^ and current findings that a specific ensemble of MePD neurons responsive to Meth and ovarian steroids are necessary for the enhancement of proceptive behaviors, but not for the display of EB + P only sexual behaviors suggests that an activation of the MePD is necessary in the enhancement of sexual motivation. Our findings are in agreement with those of Afonso *et al*., who have identified a naturally occurring variant in Long–Evans rats in which females exhibit “super-solicitational” behaviors^32^,^43^. These super- solicitational rats have a 2.5-fold increase in Fos-positive cells in the MePD compared to normal rats^32^, and MePD lesions abolish the expression of the solicitation behaviors^32^. The mechanisms mediating the increase in the activation of the MePD neurons in the Long-Evans variant are not clear. However, in our model of Meth-facilitated proceptive behaviors, signaling via the dopamine D1 receptor and progesterone receptor in the MePD are both required for the Meth-induced increase in female sexual behaviors^10^. Future work using the cFos-lacZ rats will examine whether the dopamine and progesterone signaling component requires separate MePD cell populations.

In summary, our current findings in a rodent model suggest that Meth enhances female sexual motivation via enhanced activation of a discrete neuronal population in the MePD. Taken together with our previous work, these findings suggest that this specific neuronal ensemble may mediate dopamine and progesterone signaling, both of which are necessary for the Meth-induced facilitation of proceptive behaviors. Moreover, the discovery that Meth-induced increases in proceptivity requires a specific neuronal ensemble, which is highly responsive to Meth and ovarian steroids, sets the foundation for a better understanding of the neurobiology that links sexual motivation and Meth use. Such an understanding may ultimately lead to the development of more effective therapeutic strategies to reduce high-risk sexual behaviors.

## Additional Information

**How to cite this article**: Williams, K. M. and Mong, J. A. Methamphetamine and Ovarian Steroid Responsive Cells in the Posteriodorsal Medial Amygdala are Required for Methamphetamine-enhanced Proceptive Behaviors. *Sci. Rep.* **7**, 39817; doi: 10.1038/srep39817 (2017).

**Publisher's note:** Springer Nature remains neutral with regard to jurisdictional claims in published maps and institutional affiliations.

## Supplementary Material

Supplementary Figure
