# Supplementary material for: Methamphetamine and Ovarian Steroid Responsive Cells in the Posteriodorsal Medial Amygdala are Required for Methamphetamine-enhanced Proceptive Behaviors
Source: Sci Rep. 2017 Jan 3;7:39817. doi: 10.1038/srep39817 (PMC5206624; doi:10.1038/srep39817)
Supplement: Supplementary Figure [file srep39817-s1.pdf]

Supplementary Figure

Methamphetamine and Ovarian Steroid Responsive Cells in the Posteriodorsal Medial Amygdala are Required for Methamphetamine-enhanced Proceptive Behaviors

Katrina M. Williams[1] & Jessica A. Mong[2]*
[1]*Program in Molecular Medicine, Department of Pharmacology, University of Maryland, Baltimore, MD, USA*
[2]*Department of Pharmacology, Program in Neuroscience, University of Maryland, Baltimore, MD, USA*

*Corresponding Author: Katrina M. Williams kwilliams@umaryland.edu

Figure: DAUN02 does not produce generalized damage

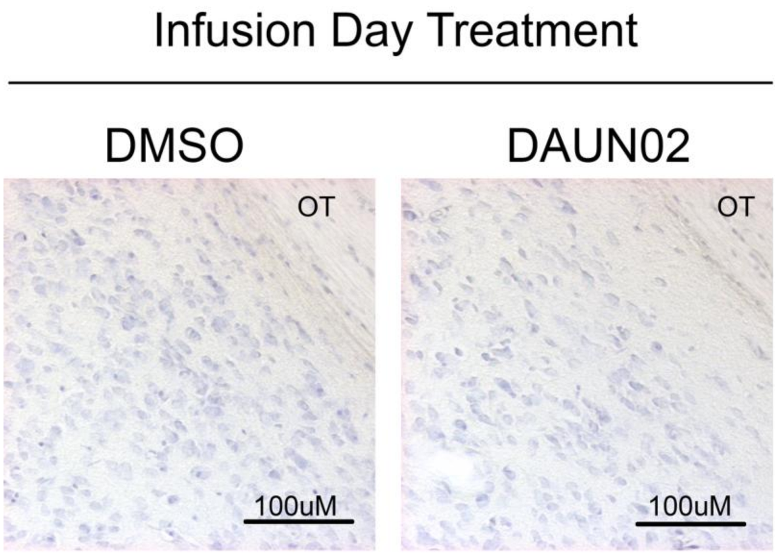

Cresyl violet staining of posteriodorsal medial amygdala obtained on final day (Day 23) of DAUN02 paradigm as part of the Confirmation Phase of c-Fos-LacZ rats. OT=optic tract.
